# Genome scale analysis of pathogenic variants targetable for single base editing

**DOI:** 10.1186/s12920-020-00735-8

**Published:** 2020-09-18

**Authors:** Alexander V. Lavrov, Georgi G. Varenikov, Mikhail Yu Skoblov

**Affiliations:** 1 Research Center for Medical Genetics, Moscow, Russia; 2 Moscow Institute of Physics and Technology, Dolgoprudny, Russia; 3 School of Biomedicine, Far Eastern Federal University, Vladivostok, Russia

**Keywords:** Base editor CRISPR/Cas9, ABE, APOBEC, PmCDA1, Pathogenic variants, Hereditary diseases

## Abstract

**Background:**

Single nucleotide variants account for approximately 90% of all known pathogenic variants responsible for human diseases. Recently discovered CRISPR/Cas9 base editors can correct individual nucleotides without cutting DNA and inducing double-stranded breaks. We aimed to find all possible pathogenic variants which can be efficiently targeted by any of the currently described base editors and to present them for further selection and development of targeted therapies.

**Methods:**

ClinVar database (GRCh37_clinvar_20171203) was used to search and select mutations available for current single-base editing systems. We included only pathogenic and likely pathogenic variants for further analysis. For every potentially editable mutation we checked the presence of PAM. If a PAM was found, we analyzed the sequence to find possibility to edit only one nucleotide without changing neighboring nucleotides. The code of the script to search Clinvar database and to analyze the sequences was written in R and is available in the appendix.

**Results:**

We analyzed 21 editing system currently reported in 9 publications. Every system has different working characteristics such as the editing window and PAM sequence. C > T base editors can precisely target 3196 mutations (46% of all pathogenic T > C variants), and A > G editors – 6900 mutations (34% of all pathogenic G > A variants).

**Conclusions:**

Protein engineering helps to develop new enzymes with a narrower window of base editors as well as using new Cas9 enzymes with different PAM sequences. But, even now the list of mutations which can be targeted with currently available systems is huge enough to choose and develop new targeted therapies.

## Background

**Fig. 1:**
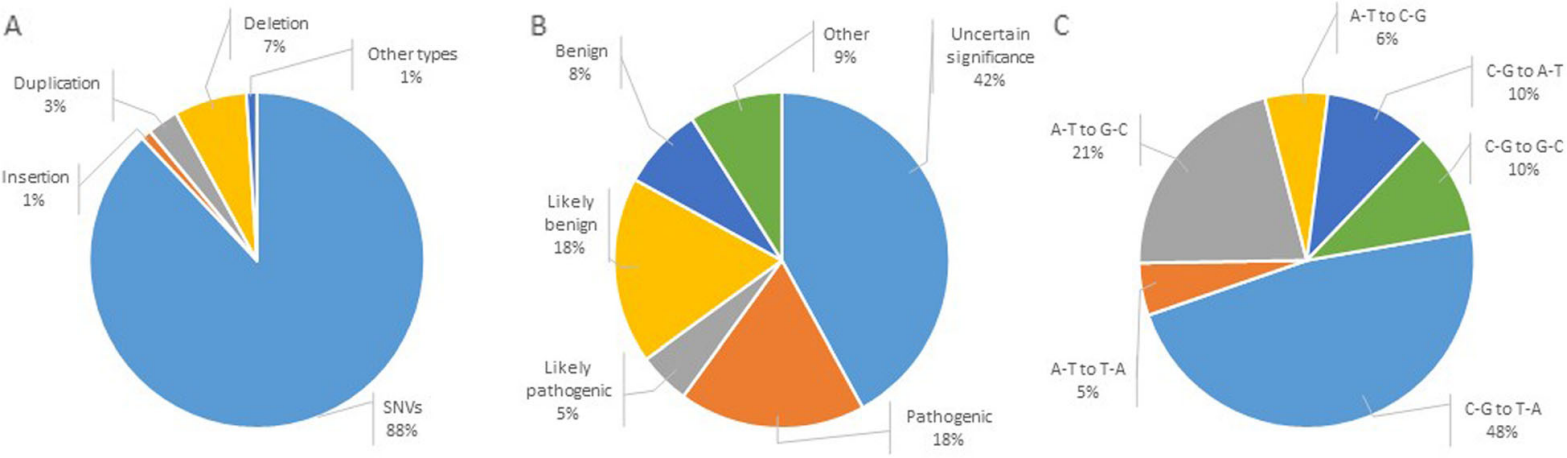
ClinVar database analysis. **a** Types of mutations. Almost 90% of them are single nucleotide variants, **b** Clinical significance of the mutations. Effects of more than 40% variants registered in ClinVar are unknown, **c** Types of SNVs in humans leading to monogenic disorders

There are currently over 6000 monogenic diseases according to OMIM [1]. Different DNA alterations may cause a disease, however the main reason of monogenic diseases is a pathogenic single nucleotide variant (SNV). SNVs account for approximately 90% of all records in ClinVar [2] database (Fig. 1a), 23% of which are pathogenic or likely pathogenic (Fig. 1b). Modern molecular genetic techniques, early diagnostics and advanced symptomatic and pathogenic treatment for many hereditary diseases are now available. Despite significant advancement in treating orphan diseases true cure is possible only by direct correction of mutated genes. Genome editing is thought to be the main breakthrough in treating monogenic diseases. The CRISPR/Cas9 system is one of the most popular tools to make changes in genome. It’s based on inducing targeted single- or double-stranded break (DSB) in DNA which is then repaired by either non-homologous end joining (NHEJ) or homology directed repair (HDR). Both approaches are used for the development of new genome editing therapeutic approaches – HDR is used to correct targeted mutations while NHEJ can be used to universally skip exons with any pathogenic mutations [3]. However all developed methods have very low efficiency with high level of unwanted events mainly due to the DSB. Moreover it was reported that DSB may be the reason of large deletions and rearrangements [4]. NHEJ is the dominating DNA repair mechanism, but it’s not precise and small insertions and deletions at the place of DSB are typical. Even in those cases when HDR successfully occurs the majority of DSBs are repaired by NHEJ.

New methods [5] can solve this problem by direct correction of individual nucleotides without inducing DSB repaired by NHEJ. CRISPR-Cas9-based single nucleotide editors developed recently may help to overcome the main obstacle in precise correction of SNVs. Their main characteristic is the direct change of the targeted nucleotide without inducing DNA breakes.

**Fig. 2:**
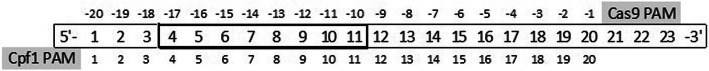
Scheme of the targeted locus with numeration of the nucleotides depending on the Cas9 or Cpf1 used in the base editor

There are two major types of base editors (BEs). Earlier developed C- > T editors are built of CRISPR-nuclease fused to cytidine deaminase [6]. Cas9/Cpf1 together with small guide RNA (sgRNA) target the construct to a specific DNA locus and cytidine deaminase converts C to T. Later developed A- > G editors use adenine deaminase. Consequently both systems depend greatly on the properties of the CRISPR protein. Cas9 has a major PAM sequence NGG placed at the 3’end of the targeted locus. Cpf1 uses PAM at the 5′-end of the sgRNA. We use numerating of the nucleotides in this work starting from the PAM: − 1, − 2, − 3… for Cas9 and + 1, + 2, + 3 for Cpf1. Both systems can typically edit nucleotides in the range of 4–11 nucleotides (− 17 – − 10 for Cas9) (Fig. 2). The width and position of the editing window depend on the properties of the deaminase and the linker between the deaminase and programmable nuclease. There are engineered nucleases with different PAMs which enlarges the number of potentially targetable DNA sequences. BEs don’t need double-stranded DNA breaks because the can successfully work with nicks of the single DNA strand. This fact is very important for the development of safe DNA-editing systems with low risk of off-target events.

Here we describe all known BEs. We also performed analysis to find all possible pathogenic variants which can be efficiently targeted by any of the described systems and present them for further selection and development of targeted therapies.

## Methods

ClinVar database (GRCh37_clinvar_20171203) was used to search and select mutations available for current single-base editing systems. We included only pathogenic and likely pathogenic variants for further analysis. Genome assembly hg19 was used as a reference.

**Fig. 3:**
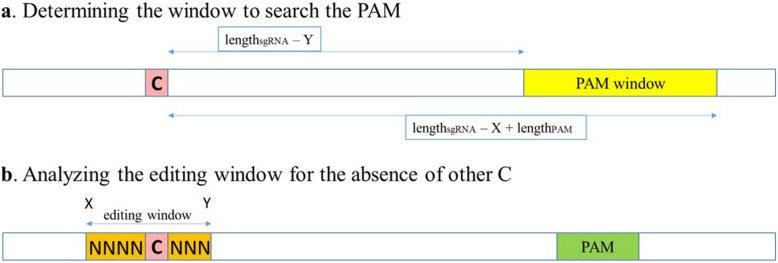
Scheme of searching for potential targets for base editors. First the script searches for PAM near (yellow) the mutation based on the characteristics of the individual editor: PAM sequence and the editing window, in which the targeted nucleotide should fit (**a**). If the PAM is found in the necessary area, the script fixes its coordinates (green) and analyses the editing window (orange) to select only the window without other cytosine (or adenine) residues to reduce the risk of unwanted editing close to zero (**b**). a, search for PAM sequences around the mutation; X – beginning of the editing window, Y – end of the editing window. b, Analysis of the DNA sequence in the editing window around the selected mutation

Generally in order to target the specific mutation the Cas9-based system needs a PAM sequence. For every potentially editable mutation the PAM sequence should be in the interval dependent on the sgRNA length and width of the editing window of the specific BE. So the PAM sequence was searched in the window with coordinates [length_sgRNA_ – Y; length_sgRNA_ – X + length_PAM_] starting from mutation location (Fig. 3, a). Where length_sgRNA_ is typically 20 for most of the systems, length_PAM_ is typically 3 and X and Y are the coordinates of the editing window for the particular BE if to count nucleotides from the 5′ end of the sgRNA. These calculations allowed to find the PAM in such a distance from the mutations that if and when BE would be applied the mutation will be found in the editable window.

If a PAM was found, we analyzed the editing window to find sequences with only one nucleotide (mutated) which can be edited without risk of changing neighboring nucleotides (Fig. 3, b). Detailed characteristics of the analyzed BEs are presented in the Table 1.

**Table 1:** Main characteristics of base editing systems

Name	Cas protein	Deaminase	PAM	Editing window	Editing Efficiency	Reference: Pubmed ID (Author, Year)	Edited mutated nucleotides
APOBEC	dCas9	APOBEC1	NGG	−18 to −11	15–75%	27,096,365 (Komor, 2016) [6]	
SaBE3	SaCas9n	APOBEC1	NNGRRT	−15 to −9	5–65%		T > C
SaKKH-BE3	dCas9	APOBEC1	NNNRRT	−17 to −9	10–65%	28,191,901 (Kim, 2017) [7]	
EQR-Cas9	dCas9	APOBEC1	NGAG	− 17 to − 10	10–40%		
VRER-Cas9	dCas9	APOBEC1	NGCG	−19 to −10	10–35%		
VQR-Cas9	dCas9	APOBEC1	NGAN	−17 to −10	10–60%		
YE1-VQR-Cas9	dCas9	APOBEC1	NGAN	−16 to −15	10–30%		
A-BE3	dCas9	APOBEC1	NGG	−17 to −12	20–50%		
Y-BE3	dCas9	APOBEC1	NGG	−17 to −13	10–30%		
FE-BE3	dCas9	APOBEC1	NGG	−16 to −14	10–40%		
YEE-BE3	dCas9	APOBEC1	NGG	−16 to −15	5–35%		
PmCDA1	nCas9	PmCDA1	NGG	−20 to −16	6–96%	27,492,474 (Nishida, 2016) [8]	
BE-PLUS	nCas9	APOBEC1	NGG	−16 to −5	10–30%	29,875,396 (Jiang, 2018) [9]	
xCas9–BE3	xCas9	APOBEC1	NGN, GAW	−17 to −13	10–24%	29,512,652 (Hu, 2018) [10]	
dCpf1-eBE	dCpf1	APOBEC1	TTTV	8 to 13	15–30%	29,553,573 (Li, 2018) [11]	
dCpf1-eBE-YE	dCpf1	APOBEC1	TTTV	10 to 12	2–28%		
APOBEC3A-Cas9	nCas9	APOBEC3A	NGG	−16 to −12	16–48%	30,059,493 (Gehrke, 2018) [12]	
EA3A-BE3(VRQR)	xCas9	APOBEC3A	NGAN	−17 to −10	15–63%		
EA3A-BE3(xCas9)	xCas9	APOBEC3A	NGG, NGT	−17 to −13	17–35%		
BE-PAPAPAP	nCas9	APOBEC1	NGG	−16 to −15	24%	30,683,865 (Tan, 2019) [13]	
cCDA1-BE3	nCas9	CDA1	NGG	−19 to − 16	50%		
xCas9–ABE	xCas9	TadA	NGV, GAT	−17 to −13	16–69%	29,512,652 (Hu, 2018) [10]	G > A
TadA	dCas9	TadA	NGG	−17 to −12	25–75%	29,160,308 (Gaudelli, 2017) [14]	

The code of the script to search the database and to analyze the sequences was written in R and is available in the Additional file 2.

## Results

Editing systems are able to convert G(C) > A(T) and A(T) > G(C), which allows in theory to correct 68% of all mutations registered in ClinVar (A(T) > G(C) – 21% and G(C) > A(T) – 47% respectively) (Fig. 1,c). We selected only pathogenic and likely pathogenic mutations – 21% of all ClinVar records. Therefore, the total number of analyzed mutations was 27,310.

We developed the R script to analyze 21 editing system currently reported in 9 publications. Every system has different working characteristics such as the editing window and PAM sequence which are summarized in the Table 1. C > T BEs have a lot of PAMs with the most popular NGG, and editing window is in the range of − 20 to − 5. For G > A mutations there are 2 systems with NGG/NGV/GAT PAMs and typical window from − 17 to − 12.

Firstly, we searched for available PAMs near the target mutation (Fig. 2,a). Exact area of searching depends on the length of the editing window and length of the sgRNA. It was possible to find several PAMs in the designated area, which were analyzed individually. For all C > T BEs, we found 6415 potential targets which constitutes 93% of all T > C pathogenic mutations. ABE systems can edit 13,683 mutations (67% of G > A pathogenic mutations).

Then we analyzed editing windows around selected mutations to check for the presence of other C(G) or A(T) nucleotides which could be nonspecifically edited together with targeted mutations. We selected only those mutations, which have no other targets near them (Table 2). As a result, for C > T systems we select 3196 variants, it is approximately 46% of all pathogenic mutations, and 6900 mutations (34% of all pathogenic) for A > G systems.

**Table 2:** Numbers of mutations targetable by different base editors

C > T Systems	Number of mutations	Number of potential sgRNAs
A-BE3	538	655
APOBEC	397	502
APOBEC3A-Cas9	115	403
BE_PLUS	181	229
EQR_Cas9	144	152
FE-BE3	714	822
PmCDA1	566	687
SaCas9	122	122
SaKKH_BE3	424	452
VQR_Cas9	485	530
VQR_Cas9_eA3A	766	766
VRER_Cas9	28	29
Y-BE3	599	722
YEE-BE3	720	791
dCpf1-eBE	136	136
dCpf1-eBE-YE	164	164
eA3A_xCas9	164	634
xCas9_BE3	2098	3001
**Total number of mutations**	**3196**	
**A > G Systems**		
TadA	2568	3235
xCas9_ABE	6829	9638
**Total number of mutations**	**6900**	

Some of the mutations can be targeted using more than one PAM, that’s why the number of potential sgRNAs can be bigger than the number of mutations

The first successful single-base editor was presented in 2016 by A. Komor with colleagues [6]. The editor consists of nuclease-deficient Cas9 fused with APOBEC1 cytidine deaminase. Cas9 with sgRNA targets the complex to DNA. Deaminase converts any cytosine into uracil in the range of 8 nucleotides from − 18 to − 11 of the targeted sequence from PAM with the overall frequency of 37%. Uracil is later repaired to thymine. The width and exact position of the window depends on the protein structure and linker length. Uracil glycosylase inhibitor was introduced to the complex to inhibit U-to-C back conversion. And finally the authors partially restored nuclease activity to cut the strand complementary to the converted nucleotide. This editor was called third-generation base editor – BE3. Later the same authors managed to develop additional systems with different editing windows and PAM sequences by changing deaminase linker length and Cas9 enzyme [7]. They succeeded in reducing window by different mutations: − 17 to − 12 for A-BE3(R126A), − 17 to − 13 for Y-BE3(W90Y), − 16 to − 14 for FE-BE3(W90F + R126E) and − 16 to − 15 for YEE-BE3(W90Y + R126E + R132E). Also, authors analyzed new Cas9 variants with altered PAMs: NGAN (VQR-Cas9) with − 17 to − 10 window and YE1-VQR-BE3 with − 16 to − 15 window, NGAG (EQR-Cas9) with − 17 to − 10 window, NGCG (VRER-Cas9) with − 19 to − 10 window. In addition, they use Cas9 homolog from *Staphylococcus aureus* (SaCas9) with PAM NNGRRT (− 15 to − 9 window) and an engineered SaCas9 variant containing three mutations (SaKKH-Cas9) with PAM NNNRRT (− 15 to − 9 window).

K. Nishida with colleagues presented a very similar editor based on another enzyme – activation-induced cytidine deaminase (PmCDA1) and nCas9 (D10A) [8]. The main difference was the editing window from − 20 to − 16 nucleotide of the targeted sequence. System demonstrated approximately 60% editing frequency in mammalian cells, with off-target mutations in lower than 1.5%. We found that nCas9(D10A)-PmCDA can target 2544 A(T) > G(C) mutations and 566 of them may be corrected without affecting nearby nucleotides.

W. Jiang with his team made a system with the longest editing window from − 16 to − 5 [9]. In 2018 J Hu et al. described modified Cas protein (xCas9) with increased number of PAMs: NG, GAA, and GAT [10].

Not only PAM sequence but also its position relative to the targeted mutations limits the usage of BEs, especially in the AT-rich regions, which are difficult to find PAMs typical for Cas9-based systems. Cpf1 has a different PAM sequence – TTTV which is also recognized upstream from the targeted sequence unlike NGG which goes immediately after targeted DNA. Cpf1 fusion with APOBEC1 allows targeting AT-rich sequences [11]. There are 2 systems with different editing windows: dCpf1-eBE from 8 to 13 and dCpf1-eBE-YE from 10 to 12.

J. Gehrke and his team tried to develop more precise BE3-based systems depending on the nucleotides neighboring the targeted mutation with TCR > TCY > VCN hierarchy [12].

Most of the pathogenic mutations are G(C) > A(T) substitutions (47%) (Fig. 1, C). That is why adenine base editor would be of great practical importance allowing correction of almost half of all mutations. However there are no natural enzymes able to convert A(T) to G(C). By direct genetic and protein engineering adenine base editor (ABE) was developed by Gaudelli NM et al. [14]. ABE consists of adenine deaminase TadA and Cas9 protein (ABE7.10). Substitution of adenine to guanine occurs in a window from − 17 to − 12 nucleotides of the targeted sequence with a probability of 60%. ABE7.10 base editor can target 7044 G(C) > A(T) mutations in the − 17 / -12 nucleotide window. Over 2/3 of them (2568) can be specifically targeted in the regions without other A(T). With modification of Cas9 and availability of additional PAMs [9] the system managed to target almost 3 times more mutations - 6829.

The full list of all targetable mutations is available in the Additional file 1.

## Discussion

Single base editors (BE) are very promising genetic tools for safe targeted correction of single nucleotide variants. They reduce the risk of indels aroused during repairing double stranded breaks. However base editors have wide editing windows and this fact which limits their potential use in editing targeted single nucleotides. Usually each nucleotide is repeated in DNA sequence in the range of 8–10 nucleotides which is the typical window width of base editors. Though there is a significant progress in the development of new BE with narrow editing windows [11] unfortunately, none of the BEs is ideally specific. Even recently developed highly specific editors claimed by the authors to edit 1–2 nucleotides at some tested loci still have a window of several nucleotides edited at very low frequency [13]. It means that if there are several targets in the window, the enzyme can edit all of them, but not only the desired target. It’s reasonable to select the most safe targets for possible genome editing with BE especially for the development of treatment in vivo. Therefore we analyzed editing windows around selected mutations to select only those which can be edited absolutely safely.

We demonstrated that about 37% of all pathogenic and likely pathogenic single nucleotide variants can be safely edited without chances to convert neighbor nucleotides. These mutations are found in 2364 genes and are responsible for the development of 4000 diseases or syndromes (based on MedGen https://www.ncbi.nlm.nih.gov/medgen/). It’s interesting to note, that 779 mutations can be edited by more than 3 analyzed BEs, which opens great potential for optimizing editing protocols.

For example one pathogenic variant NM_001005463.2:c.196A > G described in ataxia with delayed development (OMIM 617330) can be targeted by 13 different systems with 17 different sgRNAs (Table 3).

**Table 3:** Possible base editing systems to correct pathogenic variant NM_001005463.2:c.196A > G responsible for ataxia with delayed development

Base editor	Editing window	Protospacer genome sequence
A-BE3	attgga	gaaat**T**ggatttccggaggttgg
Y-BE3	attgg	gaaat**T**ggatttccggaggttgg
FE-BE3	ttg	gaaat**T**ggatttccggaggttgg
YEE-BE3	tt	gaaat**T**ggatttccggaggttgg
VQR_Cas9	ttggattt	aaat**T**ggatttccggaggttggaa
YE1-VQR-Cas9	tg	aaat**T**ggatttccggaggttggaa
APOBEC	aagaaatt	ggaagaaat**T**ggatttccggagg
APOBEC	aatTggat	gaaat**T**ggatttccggaggttgg
BE_PLUS	ggaagaaatTgga	agtggaagaaat**T**ggatttccgg
BE_PLUS	agaaatTggattt	ggaagaaat**T**ggatttccggagg
xCas9_BE3	attgg	gaaat**T**ggatttccggaggttgg
xCas9_BE3	ttgga	aaat**T**ggatttccggaggttgga
xCas9_BE3	tggat	aat**T**ggatttccggaggttggaa
APOBEC3A-Cas9	ttgga	gaaat**T**ggatttccggaggttgg
eA3A_xCas9	attgg	gaaat**T**ggatttccggaggttgg
SaKKH_BE3	gaagaaatt	gtggaagaaat**T**ggatttccggaggt
PmCDA1	ttgga	t**T**ggatttccggaggttggaagg

The non-mutated T in the genome is highlighted in bold capital letter. Since the real BE converts C > U > T, all A > G mutations were also converted to complementary sequences and algorithm was applied to the complementary sequence (containing C as a mutation but not G) if necessary. That is why the table contains only “T”s as reference nucleotides. Despite big difference in the editing length none of the windows contains Cytosine, which could be unintentionally edited together with T > C (A > G).

## Conclusions

CRISPR/Cas9 base editors allow to precisely target 46% of all T > C pathogenic mutations and 34% of all G > A pathogenic mutations. Protein engineering helps to develop new enzymes with even narrower window of editing which makes the editors more precise. Newly engineered Cas9 enzymes recognize various PAM sequences**.** Additionally the linker length between Cas9 and deaminase may help to shift the editing window to further widen the capabilities of base editors. However, even now the list of mutations which can be targeted with currently available systems is huge and allows to choose and to develop new targeted genome editing therapies.

## Supplementary information

**Additional file 1.** Table with the full list of all targetable pathogenic variants.

**Additional file 2.** Code of the script.

## Data Availability

All data generated or analyzed during this study are included in this published article and its supplementary information files.
